# Identification of *BRCA1/2* Founder Mutations in Southern Chinese Breast Cancer Patients Using Gene Sequencing and High Resolution DNA Melting Analysis

**DOI:** 10.1371/journal.pone.0043994

**Published:** 2012-09-07

**Authors:** Ava Kwong, Enders Kai On Ng, Chris Lei Po Wong, Fian Bic Fai Law, Tommy Au, Hong Nei Wong, Allison W. Kurian, Dee W. West, James M. Ford, Edmond Siu Kwan Ma

**Affiliations:** 1 Department of Surgery, The University of Hong Kong, Hong Kong SAR; 2 Department of Molecular Pathology and Department of Surgery, Hong Kong Sanatorium and Hospital, Hong Kong SAR; 3 Hong Kong Hereditary Breast Cancer Family Registry, Hong Kong SAR; 4 Departments of Medicine and Health Research and Policy, Stanford University School of Medicine, Stanford, California, United States of America; Ohio State University Medical Center, United States of America

## Abstract

**Background:**

Ethnic variations in breast cancer epidemiology and genetics have necessitated investigation of the spectra of *BRCA1* and *BRCA2* mutations in different populations. Knowledge of *BRCA* mutations in Chinese populations is still largely unknown. We conducted a multi-center study to characterize the spectra of *BRCA* mutations in Chinese breast and ovarian cancer patients from Southern China.

**Methodology/Principal Findings:**

A total of 651 clinically high-risk breast and/or ovarian cancer patients were recruited from the Hong Kong Hereditary Breast Cancer Family Registry from 2007 to 2011. Comprehensive *BRCA1* and *BRCA2* mutation screening was performed using bi-directional sequencing of all coding exons of *BRCA1* and *BRCA2*. Sequencing results were confirmed by in-house developed full high resolution DNA melting (HRM) analysis. Among the 451 probands analyzed, 69 (15.3%) deleterious *BRCA* mutations were identified, comprising 29 in *BRCA1* and 40 in *BRCA2*. The four recurrent *BRCA1* mutations (c.470_471delCT, c.3342_3345delAGAA, c.5406+1_5406+3delGTA and c.981_982delAT) accounted for 34.5% (10/29) of all *BRCA1* mutations in this cohort. The four recurrent *BRCA2* mutations (c.2808_2811delACAA, c.3109C>T, c.7436_7805del370 and c.9097_9098insA) accounted for 40% (16/40) of all *BRCA2* mutations. Haplotype analysis was performed to confirm 1 *BRCA1* and 3 *BRCA2* mutations are putative founder mutations. Rapid HRM mutation screening for a panel of the founder mutations were developed and validated.

**Conclusion:**

In this study, our findings suggest that *BRCA* mutations account for a substantial proportion of hereditary breast/ovarian cancer in Southern Chinese population. Knowing the spectrum and frequency of the founder mutations in this population will assist in the development of a cost-effective rapid screening assay, which in turn facilitates genetic counseling and testing for the purpose of cancer risk assessment.

## Introduction

The incidence of breast cancer in Asia has rapidly increased over the past 10 years and is one of the highest in Hong Kong population [1]. Several reports have found differences in breast cancer epidemiology between Asian and Caucasian populations, probably due to interactions between different lifestyle and genetic characteristics [2], [3], [4]. As the breast cancer genetic predisposition is increasingly understood, it transpires that ethnic differences exist. To date, studies of *BRCA* mutation spectrum in Chinese populations are limited [5], [6] and most of these studies were performed in single institutions or a small number of medical centers [7], [8], [9]. Some studies only focused on *BRCA1* [10], [11] and most did not perform *BRCA1/2* mutational screening using conventional full gene sequencing [12], [13]. Thus, comprehensive *BRCA1* and *BRCA2* mutation screening is rarely reported. In additional to *BRCA* mutation spectrum, identification of founder mutations in various ethnic groups is also important to improve genetic screening and cancer risk assessment because it makes a more specific approach for molecular testing that targets the founder allele possible, thus resulting in reduced cost of genetic testing and faster turnaround time. The high frequency of founder mutations in a given population provides a large patient cohort not only for robust information regarding penetrance but also accurate assessment of the effectiveness of preventive measures.

The Hong Kong Special Administrative Region of the People’s Republic of China is an advantageous location to conduct such studies related to hereditary cancers in the Chinese population where 95% of the population is comprised of Chinese [14]. Moreover, the one child policy in Mainland China that started in 1979 thus limiting the number of relatives available for genetic studies within Mainland China, does not apply to Hong Kong [15]. Previously, we reported the first Hong Kong multi-center study that comprised of 119 breast cancer patients from this region to screen for coding sequence changes in the *BRCA1* and *BRCA2* genes using conventional full gene DNA sequencing and identified a recurrent mutation c.3109C>T [16]. Although sequencing technology is still the gold standard method for mutation detection, the development of alternative methods such as high resolution DNA melting (HRM) for mutation scanning is emerging. We here set out to determine the spectrum of *BRCA1* and *BRCA2* mutations in a group of 651 Chinese probands (inclusive of the 119 probands) from Southern China using full gene sequencing and full high resolution DNA melting (HRM) analysis. Our results also prompted us to investigate the usefulness of rapid HRM for screening of the *BRCA1* and *BRCA2* founder mutations in Chinese population.

## Materials and Methods

### Ethics Statement

The study was performed in accordance with the Declaration of Helsinki. Written informed consent was obtained from all participants involved in this study. This study was approved by the Institutional Review Board of the University of Hong Kong/Hospital Authority West Cluster and other contributing hospitals, Hong Kong.

**Table 1:** Spectrum of *BRCA* pathogenic mutations identified.

Gene	Exon/Intron	Mutation Detected	AA Change	Mutation Type	No. ofCase	BIC Entries
***BRCA1***						
	IVS5	c.212+1G>T	p.C64X	SS	1	4
	5	c.212+1G>A	p.Phe46_Arg71del26		1	None
	**8**	**c.470_471delCT**	**p.Ser157X**	**FS**	**4**	**4**
	8	c.442_444delCAG	p.Gln148del		1	None
	11	c.959_960delGA	p.Arg320MetfsX5	FS	1	None
	**11**	**c.981_982delAT**	**p.Cys328X**	**FS**	**2**	**7**
	11	c.1016delA	p.K339RfsX2	FS	1	1
	11	c.1877_1878insTAGT	p.Val627SerfsX4		1	14
	11	c.1961_1962insA	p.Tyr655ValfsX18	FS	1	9
	11	c.2338C>T	p.Gln780X		1	36
	11	c.2635G>T	p.Glu879X	NS	1	2
	11	c.3214delC	p.Leu1072X		1	None
	**11**	**c.3342_3345delAGAA**	**p.Glu1115X**		**2**	**None**
	11	c.3858_3861delTGAG	p.Ser1286ArgfsX20	FS	1	1
	11	c.4049_4050insG	p.Glu1352GfsX4	FS	1	None
	11	c.4065_4068delTCAA	p.Asn1355LysfsX10	FS	1	132
	14	c.4372C>T	p.Gln1458X	NS	1	1
	15	c.4656C>G	p.Tyr1552X		1	None
	16	c.4695_4696insA	p.Ser1566IlefsX8	FS	1	None
	16	c.4903G>T	p.Glu1635X		1	None
	17–20	c.4987_5277del291	p.M1663I	IFD	1	None
	19	c.5193+1G>C	p.Trp1718SerfsX2	FS	1	None
	**22**	**c.5406+1_5406+3delGTA**	**p.Asp1778GlyfsX27**	**FS**	**2**	**None**
***BRCA2***						
	3	c.250C>T	p.Gln84X		1	5
	10	c.1261C>T	p.Q421X	NS	1	1
	11	c.2595delA	p.Glu866LysfsX8	FS	1	None
	**11**	**c.2808_2811delACAA**	**p.Ala938ProfsX21**	**FS**	**2**	**128**
	**11**	**c.3109C>T**	**p.Q1037X**	**NS**	**10**	**5**
	11	c.3202delG	p.Val1068TyrfsX9	FS	1	None
	11	c.3836delA	p.Asn1279MetfsX5	FS	1	None
	11	c.4121delA	p.Lys1374ArgfsX14	FS	1	None
	11	c.4563_4564delGT	p.L1522G*fs*X6	FS	1	None
	11	c.5164_5165delAG	p.S1722Y*fs*X4	FS	1	2
	11	c.5218_5223delTTAAGT	p.Leu1740_Ser1741del		1	None
	11	c.5722_5723delCT	p.L1908R*fs*X2	FS	1	42
	11	c.5851_5854delAGTT	p.S1951W*fs*X11	FS	1	11
	11	c.6096_6097insT	p.Ile2033TyrfsX16	FS	1	None
	13	c.7007G>T	p.Gly2313AlafsX31	FS	1	1
	14	c.7409_7410insT	p.Thr2471HisfsX4	FS	1	None
	**15–16**	**c.7436_7805del370**	**p.Asp2479GlyfsX46**	**FS**	**2**	**None**
	15	c.7467_68insT	p.I2490Y*fs*X7	NS	1	None
	15	c.7471C>T	p.Q2491X	NS	1	1
	17	c.7878G>A	p.Trp2626X		1	None
	17	c.7976+1G>A	p.Ala2603_Arg2659del57	SS	1	1
	18	c.8047_8054dupGCAAAAAC	p.L2686E*fs*X10	FS	1	None
	18	c.8066_8067delGT	p.V2690F*fs*X2	FS	1	None
	19–20	c.8332_8632del301	p.Ile2778LysfsX13	FS	1	None
	21	c.8633_8754del122	p.E2878G*fs*X5	FS	1	None
	**23**	**c.9097_9098insA**	**p.Thr3033AsnfsX11**	**FS**	**2**	**24**
	25	c.9393delC	p.Lys3132AsnfsX6	FS	1	None
	27	c.10150C>T	p.Arg3384X		1	None

***Abbreviation*** *: SS, Splice-site mutation; NS, Nonsense mutation; FS, Frame-shift mutation; IFD, In-frame deletion mutation. Recurrent mutations are highlighted in bold.*

### Patients and Selection Criteria

A total of 651 clinically high-risk breast and/or ovarian cancer patients (probands), referred to the Hong Kong Hereditary and High Risk Breast Cancer Programme (www.HRBCP.org) from 1 March 2007 to 28 Feb 2011, were recruited. This group of 651 probands contained all 119 probands from our previous report [16], in which the recurrent mutation c.3109C>T was identified. The first set of 451 patients was analyzed by full gene sequencing and HRM assays. Among the 451 patients, 24 patients were selected to use for HRM blind validation. The remaining 200 patients were recruited for recurrent mutation HRM screening only. Based on the lower incidence of breast cancer in Asia cohorts, clinically high-risk female patients who were included in this study were defined as those who: (1) had at least one first- or second- degree relative with breast and/or ovarian cancer, regardless of age; (2) were less than 50 years of age at diagnosis; (3) had bilateral breast cancer; (4) had triple negative (TN) or medullary type pathology; (5) had at least one relative with cancers other than breast and ovarian cancer such as stomach and prostate that are known to be related to *BRCA* mutations; or (6) they were ovarian cancer patients with a family history of breast cancer. The distribution of the 651 patients into the 6 categories was shown in Table S1. A standard epidemiological questionnaire, including a detailed family history, was administered to patients and medical information, including pathology reports, was retrieved from the patient’s medical records. Information from the epidemiological questionnaire included age at breast cancer diagnosis, other cancers diagnosed in the patient, and a family history of breast, ovarian, and other cancers in first, second, and third degree relatives. In addition, the following were categorized as having been used or not used: alcohol; tobacco; contraceptive pills, patches or injections; hormone replacement treatments; and infertility medications. Women were also asked if they had ever been pregnant and breast fed any child and if they were pre- or post-menopausal. Eligible patients were offered *BRCA* counseling and testing, and were consented for genetic testing and blood and tumor collection. Patients who tested positive for a *BRCA* mutation were asked to help recruit their first-degree relatives, who were also offered testing. Written informed consent was obtained from all participants involved in this study. This study was approved by the Institutional Review Board of The University of Hong Kong/Hospital Authority West Cluster and other contributing hospitals, Hong Kong.

**Table 2:** Comparisons between HRM screening and sequencing of *BRCA1* and *BRCA2* genes.

	*BRCA1*	*BRCA2*
Number of Amplicons per Patient	38	63
Number of Patients	24	24
Total Number of Reactions	912	1512
Types of Heterozygous Variants	12	16
False Positive (FP)	11	24
False Negative (FN)	0	0
True Positive (TP)	120	76
True Negative (TN)	781	1412
Sensitivity	100.0%	100.0%
Specificity	98.6%	98.3%

### *BRCA* Mutation Screening by Conventional DNA Sequencing

*BRCA1* and *BRCA2* mutation detection was performed on genomic DNA extracted from peripheral blood samples using Qiagen DNA Mini blood Kit (Qiagen, Hilden, Germany) according to manufacturer instructions. Mutation analysis was performed by direct DNA sequencing of all coding exons of *BRCA1* and *BRCA2* and partial flanking intronic sequences. PCR conditions and primer sequences are available upon request [16]. Bi-directional sequencing was performed using BigDye Terminator v3.1 Cycle Sequencing Kit and analyzed on an ABI 3130xl genetic analyzer (Applied Biosystems, Foster City, CA). Sequencing results were compared with the reference DNA sequences using Variant Reporter software (Applied Biosystems) and then reviewed manually. Computational analysis for potential cryptic splice site mutation was performed using splice site prediction programs such as NNSPLICE and ESEF finder when sequence changes were identified. All mutation and sequence variants are named according to the recommendations for the description of sequence variants of Human Genome Variation Society (HGVS). DNA sequencing was supplemented by multiplex ligation dependent probe amplification (MLPA) to detect large deletions or rearrangements [17]. However for the specific aim of validating HRM only in this study, data from DNA sequencing only was utilized.

**Table 3:** Characteristics of all probands with *BRCA1*/*2* recurrent/founder mutations, with other mutations and without mutation (N = 451).

	Patients with recurrent/founder mutations	Patients with other mutations	Patients without mutations
No. of cases	17	42	392
Gender	F = 13	F = 39	F = 366
	M = 4	M = 3	M = 26
Ethnicity	GD = 69%	GD = 93.3%	GD = 84.9%
	(9/13, missing cases = 4)	(28/30, missing cases = 12)	(275/324, missing cases = 68)
Mean age at BC diagnosed	All: 48	All: 43	All: 44
	F: 45	F: 42	F: 43
	M: 59	M: 57	M: 62
History of OC	3/13	6/39	7/366
Mean age at OC diagnosed	48	51	34
	(Range: 46–50)	(Range: 38–64)	(Range: 19–49)
No. of family member with BC	22	50	180
Avg. number of family memberwith BC in each proband	1.29	1.19	0.46

*Abbreviations: F, Female; M, Male; GD, Guangdong; N/A, Not available; BC, Breast cancer; OC, Ovarian cancer;*

### High Resolution DNA Melting Analysis

To cover all exons of *BRCA1* and *BRCA2*, 41 PCR reactions for *BRCA1* and 63 PCR reactions for *BRCA2* were developed in our HRM run per each patient sample. Thus, samples were amplified in 384-well plates. LightCycler 480 High Resolution Master kit (Roche, Penzberg, Germany) was used for HRM analysis in patient samples in LightCycler 480 system (Roche). In brief, each reaction was performed in a final volume of 10 µl containing 20 ng of DNA, 0.25 µM of each primer (forward or reverse) and 1x LightCycler 480 HRM Master mix (Roche). The PCR profile was pre-activation at 95°C for 10 min, followed by 45 cycles of 95°C for 10 sec, a touchdown from 65–53°C for 30 sec at 2.5°C/sec and 72°C for 20 sec. At the end of the PCR cycles, PCR products was denatured at 95°C for 1 min and rapidly cooled to 40°C for 1 min. HRM analyses were performed from 60°C through to 98°C at a temperature gradient of 1°C/sec, acquiring 25 data points per °C. Each sample was run in duplicates for analysis. The analytical methods have been applied previously to our mutation scanning [18]. All HRM primer sequences for *BRCA1* and *BRCA2* genes were listed in Table S2.

**Figure 1:**
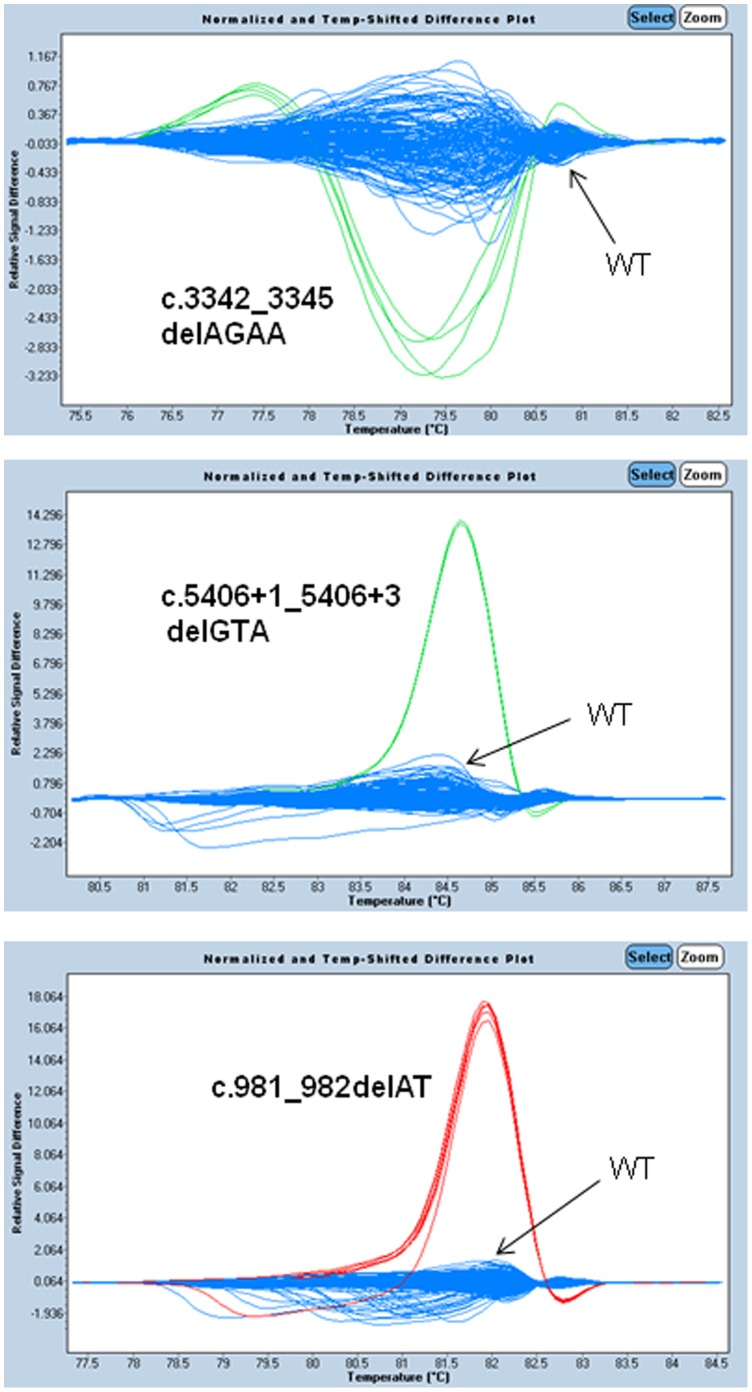
Difference plot showing the *BRCA1* recurrent or founder mutations relative to the wild type controls. The melting profile of a wild type (WT) control is chosen as a horizontal base line and the relative differences in the melting of other samples are plotted relative to this baseline. Each trace represents the amplicon from a different individual's DNA sample. Melt curves of the *BRCA1* founder mutations (green/red) were plotted against melt curve of the wild type (blue).

**Figure 2:**
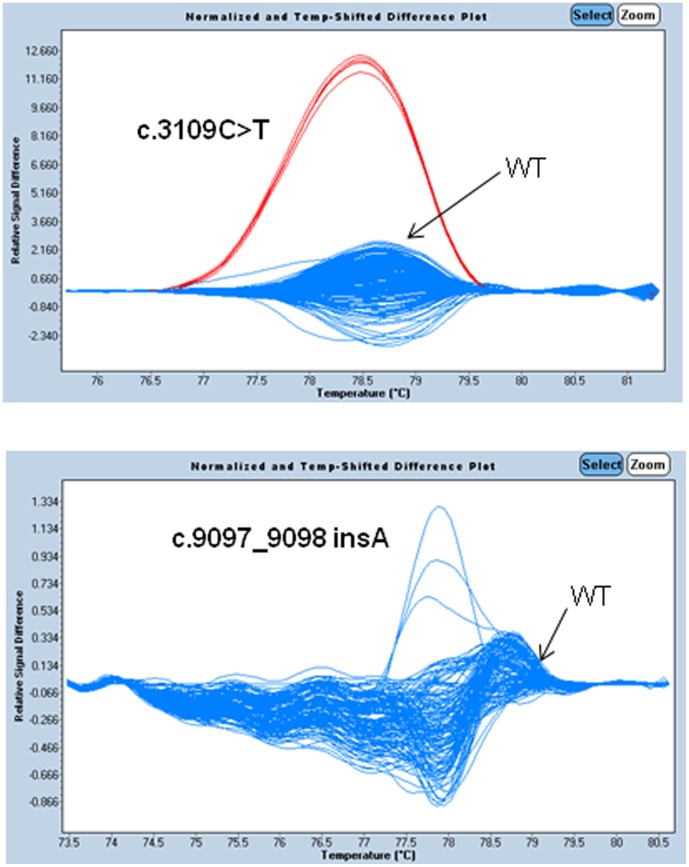
Difference plot showing the three *BRCA2* founder mutations relative to the wild type controls. The melting profile of a wild type (WT) control is chosen as a horizontal base line and the relative differences in the melting of other samples are plotted relative to this baseline. Each trace represents the amplicon from a different individual's DNA sample. Melt curves of the *BRCA2* founder mutations (green/red) were plotted against melt curve of the wild type (blue).

**Table 4:** HRM PCR primer sequences for the *BRCA* recurrent or founder mutations.

Mutations	Forward Primer Sequence (5′ to 3′)	Reverse Primer Sequence (5′ to 3′)
*BRCA1* (c.3342_3345 delAGAA)	*M13F-TTAAAGAAGCCAGCTCAAGC	#M13R-CTGAAATCAGATATGGAGAG
*BRCA1* (c.5406+1_5406+3 delGTA)	M13F-TCCCATTGAGAGGTCTTGCT	M13R-GAGAAGACTTCTGAGGCTAC
*BRCA1* (c.981_982delAT)	M13F-TCATGCCAGCTCATTACAGC	M13R-TCAGACTCCCCATCATGTGA
*BRCA2* (c.3109C>T)	M13F-AAATGGGCAGGACTCTTAGGT	M13R-CTACACTACTCTGTAAATGTGC
*BRCA2* (c.9097_9098 insA)	M13F-TTTAAATGATAATCACTTCTTCC	M13R-TCCATAAACTAACAAGCACTTAT

* M13F: TGTAAAACGACGGCCAGT.

# M13R: CAGGAAACAGCTATGACC.

### Haplotype Analysis

Individuals with identical *BRCA1* and *BRCA2* germline mutations from unrelated families were genotyped for allele sharing indicative of a common ancestor. Thus, haplotype analysis was conducted at 6 microsatellite polymorphic loci D17S791, D17S855, D17S1323, D17S1322, D17S1335 and D17S1185 of the *BRCA1* gene and at 6 loci D13S289, D13S1699, D13S1698, D13S171, D13S1695 and D13S260 of the *BRCA2* gene. Primer sequences of all microsatellite polymorphic markers were listed in Table S3. These markers were localized in a ∼5 Mb region encompassing *BRCA1* on chromosome 17q21.2–17q21.31 and a ∼2.5 Mb region encompassing *BRCA2* on chromosome 13q12.3–13q13.1. Fluorescently end-labeled primers were used to amplify the microsatellite polymorphic regions. Size fractioning of PCR products were performed on a 3130xl Genetic Analyzer (Applied Biosystems) using the GeneScan 500 LIZ Size Standard and analyzed using the GeneMapper v3.7 software (Applied Biosystems). Haplotype estimations were performed using software program PHASE.

### Statistical Analysis

P values from χ^2^ analyses describe any difference in *BRCA* mutations carriers among all Chinese female patients. The significance of age and *BRCA* status of patients was determined by Wilcoxon test. Fisher's exact test was used in the analysis of categorical data where expected counts are less than 5. Linear-by-Linear association was used in the analysis of ordinal data. SPSS for Windows Release 16.0 (SPSS Inc, Chicago, USA) was used to analyze the data; statistical significance and marginal statistical significance were set at *P*<0.05 and *P*<0.1 respectively.

## Results

### Patient Characteristics

This study included 651 probands (616 female and 35 male), comprised of 611 breast cancer patients,17 ovarian cancer patients and the remaining 23 patients with both breast and ovarian patients. The mean age at diagnosis of breast cancer was 43 years old (range 18–82) and that of ovarian cancer was 43 years old (range 19–64). All probands were from Chinese ancestry and over 90% were from Guangdong province of Southern China.

### Identification of *BRCA1* and *BRCA2* Mutations Using Bi-directional Sequencing

Extensive sequence analysis of all coding exons in *BRCA1* and *BRCA2* of a total of 451 probands out of the 651 patients were conducted. Based on our sequencing results, 69 (69/451, 15.3%) deleterious *BRCA* gene mutations were identified. Of the 69 deleterious mutations, 29 (29/69, 42%) were in *BRCA1* and 40 (40/69, 58%) in *BRCA2*. There was no significant difference in the age of breast cancer diagnosis between *BRCA* carriers and non-*BRCA* carriers (*p* = 0.325, Wilcoxon test). Although the mean age of breast cancer diagnosis of *BRCA2* mutation carriers (mean age 45.7) are slightly higher than that of *BRCA1* mutation carriers (mean age 40.4), the difference was not significant (*p* = 0.455, Wilcoxon test). The spectra of all deleterious mutations identified are illustrated in Table 1. Of the 69 deleterious mutations, we identified 29 (42.0%) novel deleterious mutations that have not been published in the Breast Information Core Database of National Institute of Health (BIC) as shown in Table 1. Among the 29 novel mutations, 12 were in *BRCA1* and 17 were in *BRCA2*. Most of the novel deleterious mutations cause sequence frame-shift, leading to early termination of translation for protein products. In this study, we identified 4 recurrent *BRCA1* mutations (c.470_471delCT, c.3342_3345delAGAA, c.5406+1_5406+3delGTA and c.981_982delAT) accounted for 34.5% (10/29) of all *BRCA1* mutations and 4 recurrent *BRCA2* mutations (c.2808_2811delACAA, c.3109C>T, c.7436_7805del370 and c.9097_9098insA) accounted for 40% (16/40) of all *BRCA2* mutations. In addition, among our cohort of the 33 male probands, 7 (7/33, 21.2%) deleterious mutations were found. Intriguingly, all 7 male probands carried *BRCA2* deleterious mutations only.

### Diagnostic Performance of In-house Developed Full HRM for Screening of *BRCA1* and *BRCA2* Mutations

Apart from the development of full *BRCA1* and *BRCA2* gene sequencing, we also developed full HRM assays for rapid screening of *BRCA1* and *BRCA2* mutations. In our developed full gene HRM assays, there are 38 *BRCA1* and 63 *BRCA2* HRM reactions per patients were established. In order to validate the testing performance of our in-house developed HRM assays, 8 probands with known *BRCA1* deleterious mutations and 12 probands with known *BRCA2* deleterious mutation from the 451 patients were analyzed. Those known *BRCA* mutations were previously identified by our full gene sequencing. In this first validation set, all 24 deleterious mutations and variants identified by sequencing were also detected by our in-house developed HRM assays. Thus, the detection rate for variant detection was 100% when compared to sequencing.

In the second blind validation set, a cohort of 24 breast cancer patients from the 451 study patients was analyzed. Of all 24 patients, there were 2 patients with different *BRCA1* deleterious mutations and 2 patients with different *BRCA2* deleterious mutations. The detailed breakdown of all mutations and variants in the 24 patients was shown in Table S4. The results of the mutation sequencing were blinded to the operator until all samples had been scored by HRM assays. A total of 912 *BRCA1* and 1512 *BRCA2* amplicons were analyzed (Table 2). All deleterious mutations examined by sequencing were also detected by HRM. However, the HRM false positive rates of *BRCA1* and *BRCA2* were 1.2% (11/912) and 1.6% (24/1512) respectively. The calculated sensitivity and specificity for *BRCA1* mutation and variant detection was 100% and 98.6% respectively while the sensitivity and specificity for *BRCA2* mutation and variant detection was 100% and 98.3% respectively (Table 2).

### Founder Mutations Confirmed by Haplotype Analysis

In this study, a total of 4 recurrent *BRCA1* mutations (c.470_471delCT, c.3342_3345delAGAA, c.5406+1_5406+3delGTA and c.981_982delAT) and 4 recurrent *BRCA2* mutations (c.2808_2811delACAA, c.3109C>T, c.7436_7805del370 and c.9097_9098insA) were identified in our Chinese cohort (Table 1). To determine whether these recurrent mutations have arisen from a common ancestor, haplotype analysis was performed in unrelated families including 10 *BRCA1* probands with 20 of their family members, 12 *BRCA2* probands with 27 of their family members and 50 unrelated Chinese individuals without any *BRCA* mutations. For *BRCA1* recurrent mutations, the genotypes of all probands, family members and unrelated controls were examined at 6 polymorphic markers (3 are *BRCA1* intragenic markers). For *BRCA2*, the genotypes of all cases were examined at 6 polymorphic markers (Table S5). Haplotype analysis for each recurrent mutation contained at least two unrelated families in which at least one of them has available family members. Our results revealed that except for the mutation negative family members and the 50 unrelated controls, carriers with the recurrent *BRCA1* mutation (c.981_982delAT) and 3 recurrent *BRCA2* mutations (c.3109C>T, c.7436_7805del370 and c.9097_9098insA) shared the same haplotype suggesting that these 4 putative founder mutations are derived from a common ancestor (Tables S5). For the previously confirmed *BRCA2* mutation (c.3109C>T) [16], 6 additional unrelated patients were identified from this cohort. Together with previous patients identified, this founder mutation (c.3109C>T) accounted for 25% (10/40) of all *BRCA2* mutations and so far is the highest proportion found in our cohort. For the only *BRCA1* founder mutation confirmed, it accounted for 6.9% (2/29) all *BRCA1* mutations in this cohort while all the *BRCA2* founder mutations accounted for 35% (14/40) of all *BRCA2* mutations. Characteristics of all 451 probands with or without *BRCA* mutations were shown in Table 3. Based on this data, probands with *BRCA* mutations have higher frequency of the family history of ovarian cancer than those without *BRCA* mutations. Furthermore, probands with *BRCA* mutations have greater average number of family member with breast cancer than those without *BRCA* mutations.

### Development of Rapid HRM Screening Assays for Founder Mutations

As some recurrent mutations were confirmed to be founder mutations in the Southern China population, we then rapidly developed HRM screening assays targeting each founder or recurrent mutations so as to further screen our Chinese population. Due to the 370 bp deletion, *BRCA2* founder mutation (c.7436_7805del370) is not easily detected by HRM. Thus, a panel of 5 HRM assays for the remaining founders was developed (Figure 1 and 2) and the primer sequences were listed in Table 4. HRM screening assays were performed on an independent cohort of 200 Chinese breast cancer patients. Our results indicated that our developed HRM assays can rapidly detect one more patients carried *BRCA2* founder mutation (c.3109C>T) from the 200 patients. Taken all together, the overall frequency of *BRCA2* (c.3109C>T) founder mutation observed was 1.7% (11/651) among 651 Chinese patients and accounted for 26.8% (11/41) of all *BRCA2* mutations in our Southern Chinese cohort. The total of 3 recurrent *BRCA2* mutations then accounted for 36.6% (15/41) of all *BRCA2* mutations.

## Discussion

In this study, we report the contributions of *BRCA* mutations to high-risk families of Chinese population from Southern China. Increasing cancer rates are of great concern to these Asian countries because these countries often have limited health and medical care resources and infrastructures to meet the needs of these patients. Thus, it is important to obtain a better understanding of the causes of breast cancer among Asians and other populations so as to improve prevention and cancer risk assessment efficiently worldwide. To our knowledge, this is one of the larger Chinese cohort studies comprising of 651 probands and comprehensive full *BRCA1* and *BRCA2* gene sequencing on 451 probands was employed for mutation screening analysis. Our findings revealed that the proportion of *BRCA* mutation is15.3% in a defined high risk group of Chinese population while Caucasian cohorts are estimated to be 5–13% [19], [20]. As there are variations in selection criteria, such difference is difficult to be compared whether significance is obtained between Chinese and Caucasian populations.

Unlike the Caucasian population, we found that a relative predominance of *BRCA2* mutations (58%), similar to that reported in some Asian studies [9], [21], [22], [23]. This may be that breast cancer biology in Chinese is different to that of the Western population. The predominance of *BRCA2* founder mutations in our cohort was higher when compared to that of the *BRCA1* founder mutations. Of note, a similar pattern of a predominance of *BRCA2* mutations compared to *BRCA1* was recently observed in a study of high-risk Asian-Americans [24].

In this study, we identified 29 novel deleterious *BRCA* mutations which have not been published in the BIC Database of NIH. This proportion is relatively high in our Chinese cohort. Reports have found a high frequency of variants in different ethnic populations [8], [11], [25], [26]. This is likely due to the limited knowledge of the mutation spectrum in different ethnic populations where *BRCA* testing have not be widely performed, especially in Chinese population [27].

Notably, we discovered three *BRCA1* and three *BRCA2* putative founder mutations in our Southern Chinese cohort. Interestingly, we also observed that half of the putative founder mutations (3 of 6) have not been published in the BIC Database (Table 1). Since there are only two cases reported for each of the novel putative founder mutation (*BRCA1* c.3342_3345delAGAA, c.5406+1_5406+3delGTA and *BRCA2* c.7436_7805del370), their founder effects are required to be confirmed by larger sample size of unrelated probands. The *BRCA1* and *BRCA2* recurring mutations that did not share a common haplotype could also be attributable to factors such as age of mutation such that associated alleles are no longer in linkage disequilibrium with microsatellite marker. Founder mutations not only provide population-specific genetic risk assessment, but are also useful in the study of penetrance of *BRCA* mutation in a specific population [3], [28], [29], [30], [31]. Most studies reported are based on women with ovarian cancers, some did not perform haplotype studies, some cannot rule out the possibility of somatic mutations due to the use of only tumor samples [32] and others required a larger cohort to confirm its founder effect [12], [33], [34]. Further study with a large-scale Chinese population size is required to evaluate the association between this founder mutation and breast cancer risk. The finding from this study suggests that future study will provide valuable information for genetic counseling and testing in cancer risk assessment.

Based on our recurrent mutations identified in this study, some of them such as *BRCA1* c.981_982delAT and *BRCA2* c.3109C>T are one of the most common mutations found in Asian countries such as China, Korea and Singapore [34], [35], [36]. As expected, these common mutations are confirmed to be founder mutations. A number of other mutations were observed at high frequency in Korea [35], and which were at higher prevalence than those previously reported as founders. Interestingly, recurrent mutations, *BRCA1* c.5496_5506del11insA, *BRCA1* c.390C>A, *BRCA2* c.7480C>T and *BRCA2* c.3109C>T, are unique mutations that were not found in either other Asian or even Caucasian populations according to our database search from BIC and HGVS. On the other hands, one recurrent mutation *BRCA2* c.2808_2811delACAA was frequently observed in other ethnic populations such as Caucasian, African American, Hispanic and Australia.

The discovery of this founder mutation may provide a cost-effective option to rapidly screen a population. Our finding of complete concordance between conventional sequencing data and the HRM output in patients DNA suggests that the HRM technology is ready for use in diagnostic setting. Furthermore, there are several advantages of using HRM over other mutation screening methods: (i) Recent reports showed that the sensitivity and specificity of HRM is better than that of denaturing high-performance liquid chromatography (DHPLC) which is the current gold standard of scanning methods [37]; (ii) HRM is more rapid as the melting analysis is performed in all wells simultaneously i.e. a closed system whereas DHPLC or other methods such as fluorescent multiplexed-PCR analysis (FMPA) involves the post-PCR manipulations and then the sequential analysis of each sample; (iii) HRM is excellent for heterozygote detection. Without any labeled probe, HRM can differentiate between heterozygote and homozygote simultaneously with high accuracy [38]. In our study these *BRCA1* and *BRCA2* founder mutations detectable by conventional DNA sequencing were also detectable by HRM, giving a high sensitivity for the latter method. The true sensitivity remains to be determined in a larger cohort including unselected Chinese women and men.

In conclusion, we conducted an extensive *BRCA* mutation analysis in a large Southern Chinese cohort and four founder mutations were identified. We then rapidly developed HRM mutation screening assays for those recurrent or founder mutations. The only *BRCA1* confirmed founder mutations account for 6.9% of all identified *BRCA1* mutations, whereas *BRCA2* founder mutations account for 37.5% of all *BRCA2* mutations. Our findings indicate that both *BRCA1* and *BRCA2* mutations account for a substantial proportion of hereditary breast/ovarian cancer in the Southern Chinese population. Identification of a founder mutation and knowledge of its prevalence in the Southern Chinese population provides important information both to genetic counseling and cancer risk assessment, as well as to the development of a cost-effective screening strategy.

## Supporting Information

Table S1 **Distribution of the patients in this study according to the recruitment criteria.**
(DOC)

Table S2 **Sequences of high resolution melting (HRM) primers for** ***BRCA1*** **and** ***BRCA2*** **genes.**
(DOC)

Table S3 **Sequences of PCR primers for microsatellite polymorphic markers.**
(DOC)

Table S4 **The breakdown of mutations and variants of** ***BRCA1*** **and** ***BRCA2*** **in the 24 patients in the blind validation.**
(DOC)

Table S5 **Genotype of carriers with** ***BRCA1*** **or** ***BRCA2*** **founder mutations and family members without mutations.**
(DOC)
