# Supplementary material for: Identification of BRCA1/2 Founder Mutations in Southern Chinese Breast Cancer Patients Using Gene Sequencing and High Resolution DNA Melting Analysis
Source: PLoS One. 2012 Sep 7;7(9):e43994. doi: 10.1371/journal.pone.0043994 (PMC3436879; doi:10.1371/journal.pone.0043994)
Supplement: Table S1 — Distribution of the patients in this study according to the recruitment criteria. (DOC) [file pone.0043994.s001.doc]

**Table S1** Distribution of the patients in this study according to the recruitment criteria

| **Recruitment criteria** | **Gender** | **Number of patients** | | **Total** |
| --- | --- | --- | --- | --- |
|  |  | ***BRCA* negative** | ***BRCA* positive** |  |
| At least one 1^st^ or 2^nd^ degree relative with breast and/or ovarian cancer | F | 145 | 42 | 187 |
|  | M | 3 | 3 | 6 |
| Less than 50 years of age at diagnosis | F | 287 | 41 | 328 |
|  | M | 4 | 1 | 5 |
| Bilateral cancer | F | 58 | 14 | 72 |
|  | M | 0 | 0 | 0 |
| Medullary type | F | 3 | 3 | 6 |
|  | M | 0 | 0 | 0 |
| Triple negative | F | 58 | 18 | 76 |
|  | M | 0 | 0 | 0 |
| Had at least one relative with cancers other than breast and ovarian cancer | F | 109 | 6 | 115 |
|  | M | 5 | 2 | 7 |
| Ovarian cancer patients with a family history of breast cancer | F | 3 | 2 | 5 |
|  | M | 0 | 0 | 0 |
