# Supplementary material for: Identification of BRCA1/2 Founder Mutations in Southern Chinese Breast Cancer Patients Using Gene Sequencing and High Resolution DNA Melting Analysis
Source: PLoS One. 2012 Sep 7;7(9):e43994. doi: 10.1371/journal.pone.0043994 (PMC3436879; doi:10.1371/journal.pone.0043994)
Supplement: Table S2 — Sequences of high resolution melting (HRM) primers for BRCA1 and BRCA2 genes. (DOC) [file pone.0043994.s002.doc]

**Table S2** Sequences of high resolution melting (HRM) primers for *BRCA1* and *BRCA2* genes

| ***BRCA1*** | | | | | |
| --- | --- | --- | --- | --- | --- |
| ***Exon*** | ***Amplicon length*** | ***Forward Primer Sequence (5' to 3')*** | | ***Reverse Primer Sequence (5' to 3')*** | |
| 2 | 260 | ^*^M13F-GGACGTTGTCATTAGTTCTTTGG | | ^#^M13R-TCCCAAATTAATACACTCTTGTGC | |
| 3 | 311 | M13F-TTGAGGCCTTATGTTGACTCAG | | M13R-TGAAATGGAGTTGGATTTTTCG | |
| 5 | 256 | M13F-TTCATGGCTATTTGCCTTTTG | | M13R-TGATGAATGGTTTTATAGGAACG | |
| 6 | 282 | M13F-GGTTTTCTACTGTTGCTGCATCT | | M13R-GAAAGTAATTGTGCAAACTTCCTG | |
| 7 | 246 | M13F-GGGTTTCTCTTGGTTTCTTTGA | | M13R-AGAAGAAGAAAACAAATGGTTT | |
| 8 | 284 | M13F-TTCAGGAGGAAAAGCACAGAA | | M13R-CACTTCCCAAAGCTGCCTAC | |
| 9 | 181 | M13F-ACCCTTTTAATTAAGAAAACTTTTAT | | M13R-AAAGAGAGAAACATCAATCCT | |
| 10 | 282 | M13F-TGGTCAGCTTTCTGTAATCGAA | | M13R-AAGGTCCCAAATGGTCTTCA | |
| 11-1 | 348 | M13F-GTTGATTTCCACCTCCAAGG | | M13R-GTTTGCTTTTATTACAGAATTCAGCC | |
| 11-2 | 378 | M13F-TCATGCCAGCTCATTACAGC | | M13R-TCAGACTCCCCATCATGTGA | |
| 11-3 | 221 | M13F-GTGTGAGAGAAAAGAATGG | | M13R-CATCTACCTCATTTAGAACG | |
| 11-4 | 281 | M13F-GAATCAAATGCCAAAGTAGC | | M13R-GGACGCTCTTGTATTATCTG | |
| 11-5 | 284 | M13F-ATTATAGGAGCATTTGTTAC | | M13R-TTTTCGAGTGATTCTATTGG | |
| 11-6 | 236 | M13F-CAAAAGGTGATTCTATTCAG | | M13R-ATTAGGTGGGCTTAGATTTC | |
| 11-7 | 218 | M13F-CAGGCATATTCATGCGCTTG | | M13R-TACTTGTCTGTTCATTTGGC | |
| 11-8 | 297 | M13F-AGGTAAAGAACCTGCAAC | | M13R-ATACTGCTACTCTCTACAGAT | |
| 11-9 | 292 | M13F-GTGGAGAAAGGGTTTTGCAA | | M13R-CTTTCTTCCATTTCTATGCTTGT | |
| 11-10 | 311 | M13F-GTATCCATTGGGACATGAAG | | M13R-GACCAACCACAGGAAAGCCT | |
| 11-11 | 209 | M13F-CAAGCCTGTACAGACAGTTA | | M13R-TGATGGGAAAAAGTGGTGGT | |
| 11-12 | 208 | M13F-GCCAAATGTAGTATCAAAGG | | M13R-CAGGTGACATTGAATGTTCC | |
| 11-13 | 210 | M13F-AAAATCTGCTAGAGGAAAAC | | M13R-CATCACTGGAACCTATTTC | |
| 11-14 | 285 | M13F-TTAAAGAAGCCAGCTCAAGC | | M13R-CTGAAATCAGATATGGAGAG | |
| 11-15 | 240 | M13F-TGAAGAAGTAGTTCAGACTG | | M13R-AAAGGGCTAGGACTCCTGCT | |
| 11-16 | 232 | M13F-AGTCATGCATCTCAGGTTTG | | M13R-ATAAGTTCTCTTCTGAGGAC | |
| 11-17 | 237 | M13F-CTTTCACCCATACACATTTG | | M13R-TGCAGTCATTTAAGCTATTC | |
| 11-18 | 181 | M13F-GAGTGTCTGTCTAAGAACAC | | M13R-TATTTGCAGTCAAGTCTTCC | |
| 11-19 | 351 | M13F-TAATATTGGCAAAGGCATCTCA | | M13R-GCTCCCCAAAAGCATAAACA | |
| 12 | 215 | M13F-CAGCAAGTTGCAGCGTT | | M13R-ATACATACTACTGAATGCAAAGGAC | |
| 13 | 305 | M13F-AATGGAAAGCTTCTCAAAGTATT | | M13R-CCTTACTCTTCAGAAGGAGAT | |
| 14 | 312 | M13F-CTAACCTGAATTATCACTATC | | M13R-GTGTATAAATGCCTGTATGCA | |
| 15 | 310 | M13F-CTTTCACAATTGGTGGCG | | M13R-CCAGAATATCTTTATGTAGGATTCAG | |
| 16-1 | 260 | M13F-GACCAGAACTTTGTAATTC | | M13R-CCCAGCAGTATCAGTAGTAT | |
| 16-2 | 237 | M13F-AAAGTTGCAGAATCTGCCC | | M13R-CATAAAACTCTTTCCAGAATGTTG | |
| 17 | 248 | M13F-TGTAGAACGTGCAGGATTGC | | M13R-TTTATGCAGCAGATGCAAGG | |
| 18 | 206 | M13F-CTTTAGCTTCTTAGGACAGCA | | M13R-AAATGCAATTCTGAGGTGTTA | |
| 19 | 211 | M13F-TTGTGAATCGCTGACCTCTCT | | M13R-GGTGCATTGATGGAAGGAAG | |
| 20 | 230 | M13F-CTGGCCTGAATGCCTTAAAT | | M13R-CAGAGTGGTGGGGTGAGATT | |
| 21 | 191 | M13F-AGATTTTCCTTCTCTCCATTCC | | M13R-CCATCGTGGGATCTTGCTTA | |
| 22 | 297 | M13F-TCCCATTGAGAGGTCTTGCT | | M13R-GAGAAGACTTCTGAGGCTAC | |
| 23 | 189 | M13F-GATGAAGTGACAGTTCCAGTAG | | M13R-GTGATAAACCAAACCCATGC | |
| 24 | 246 | M13F-CCTAGTCCAGGAGAATGAATTGA | | M13R-CTGGAAAGGCCACTTTGTAA | |
| ***BRCA2*** | | | | | |
| ***Exon*** | ***Amplicon length*** | | ***Forward Primer Sequence (5' to 3')*** | ***Reverse Primer Sequence (5' to 3')*** | |
| 2 | 258 | | M13F-CAACACTGTGACGTACTGGGT | M13R-CTCAGTCACATAATAAGGAATGC | |
| 3-1 |  | | M13F-TGGTTAAAACTAAGGTGGGATTTT | M13R-TCAGCCCTTGCTCTTTGAAT | |
| 3-2 |  | | M13F-AAAACTCCACAAAGGAAACCA | M13R-GAGACTGATTTGCCCAGCAT | |
| 4 | 225 | | M13F-CAAAGAATGCAAATTTATAATC | M13R-ATATGTAGGAAAATGTTTCA | |
| 5 | 277 | | M13F-TTTTTAAAATAACCTAAGGGATTTG | M13R-CACATACCACTGGGGGTAAAA | |
| 6 | 251 | | M13F-CTACAATGTACACATGTAACAC | M13R-AATCTCAGGGCAAAGGTATAAC | |
| 7-1 | 126 | | M13F-ATCAGGGCATTTCTATAA | M13R-TAGCTAAAGAACTTGACC | |
| 7-2 | 134 | | M13F-TATTTCTGAAAGTCTAGGAG | M13R-ATCTGCTCTTTCTTGTAA | |
| 8 | 234 | | M13F-GTGTCATGTAATCAAATAGTAGAT | M13R-AATGTAAGATAAATAATTTAACAAGG | |
| 9 | 262 | | M13F-GGACTACTACTATATGTGCATTG | M13R-TGACAGAGCAAGACTCCACCTC | |
| 10-1 | 315 | | M13F-AAATATTAATGTGCTTCTGT | M13R-ACTTCAGATACAAATGAGT | |
| 10-2 | 304 | | M13F-ATTTTCCATGAAGCAAAC | M13R-TCTCTGTGTCTAATAGGT | |
| 10-3 | 253 | | M13F-GAGAAAATACCCCTATTG | M13R-GAGAAGTTCCAGATATTG | |
| 10-4 | 314 | | M13F-TCTTGAATCTCATACAGAC | M13R-CTGCATTCTTCAAAGCTA | |
| 10-5 | 290 | | M13F-GCCACCACCACACAGAAT | M13R-TGCAAATGTAAGTGGTGCTTC | |
| 10-6 |  | | M13F-TCGCAATGAAAGAGAGGAAGA | M13R-CAGAAGGAATCGTCATCTATAAAAC | |
| 11-1 | 243 | | M13F-GGTACTTTAATTTTGTCACTTTG | M13R-TCTGGGGTAATAAATAACTGTAG | |
| 11-2 | 230 | | M13F-CAGTAATCTCTCAGGATCTTG | M13R-CTGGGATTGAAAGTCAGTATC | |
| 11-3 | 257 | | M13F-TGCAGCATGTCACCCAGTAC | M13R-CATCTTGATTCTTTTCCATGGG | |
| 11-4 | 261 | | M13F-AGGTAACAATTATGAATCTGATG | M13R-TTGTCTGAGAAAAGTTCTTCAG | |
| 11-5 | 267 | | M13F-CAAGAAGAAACTACTTCAATTTC | M13R-TGCAAGAACATAAACCAAATCTT | |
| 11-6 | 252 | | M13F-ATGGAGACACAGGTGATAAAC | M13R-TTGAAGCTGTTCTGAAGCTAC | |
| 11-7 | 256 | | M13F-AAATGGGCAGGACTCTTAGGT | M13R-CTACACTACTCTGTAAATGTGC | |
| 11-8 | 270 | | M13F-TCAAAAGAAACTGAGCAAGCC | M13R-GGCACTTCAAATGTACTCTTC | |
| 11-9 | 289 | | M13F-AAGAATCAGGAAGTCAGTTTGA | M13R-CCCACTTCATTTTCATCTGTTA | |
| 11-10 | 240 | | M13F-TGCTGGCCTGTTGAAAAATGA | M13R-ATTGAAACAACAGAATCATGAC | |
| 11-11 | 282 | | M13F-AAACTTCTGCAGAGGTACATC | M13R-TTTTACTTGAATCACTGCCATC | |
| 11-12 | 283 | | M13F-ATAACAAATATACTGCTGCCAG | M13R-TCCGTTTTAGTAGCAGTTAACT | |
| 11-13 | 313 | | M13F-GCGAAAGCTCAAGAAGCATG | M13R-CTGGGACACTTTCTTTCAGTA | |
| 11-14 | 253 | | M13F-TGGACATTCTAAGTTATGAGGA | M13R-AAACTGGTGATTTCACTAGTAC | |
| 11-15 | 293 | | M13F-AATCTTTGGACAAAGTGAAAAAC | M13R-AAAGATACTTTTTGATGTTTTGAG | |
| 11-16 | 271 | | M13F-CCACCTAAGCTCTTAAGTGAT | M13R-GTTGACCATCAAATATTCCTTC | |
| 11-17 | 291 | | M13F-ACTTCTGTGAGTCAGACTTCA | M13R-TACTGGCTCAATACCAGAATC | |
| 11-18 | 276 | | M13F-CCTACCATTCTGATGAGGTAT | M13R-GTGGCCCTACCTCAAAATTAT | |
| 11-19 | 261 | | M13F-CTAGCTCTTCACCCTGCAAA | M13R-CCTCTGAATCATCCAATGCC | |
| 11-20 | 289 | | M13F-CGAGAATAAATCAAAAATTTGCC | M13R-AAGTATTTGCAGATGAGACTGA | |
| 11-21 | 275 | | M13F-CACCTTGTGATGTTAGTTTGG | M13R-TTCTGGAGTACGTATAGCAGT | |
| 11-22 | 280 | | M13F-AAAAGTAACGAACATTCAGACC | M13R-CAACACGAGGAAGTATTTTTGA | |
| 11-23 | 267 | | M13F-GAACTGAGCATAGTCTTCAC | M13R-TCTCAACAAGTGAGACTTTGG | |
| 11-24 | 272 | | M13F-TCTCTCTCAATTTCAACAAGAC | M13R-GTTTAGAATCTGTCAGTTCATC | |
| 11-25 | 217 | | M13F-TACTTTGAAACAGAAGCAGTAG | M13R-TAGTGATTGGCAACACGAAAG | |
| 12 | 216 | | M13F-AAAATGGTCTATAGACTTTTGAG | M13R-ACCTATAGAGGGAGAACAGAT | |
| 13 | 189 | | M13F-ACAGTAACATGGATATTCTCTTA | M13R-AAACGAGACTTTTCTCATACTG | |
| 14-1 | 310 | | M13F-TGCAACAAAGGCATATTCCTAA | M13R-GTGATTTAGTTTTAAAAGGTGG | |
| 14-2 | 302 | | M13F-TTGATTACTACAGGCAGACCA | M13R-GAAATATCTAACTGAAAGGCAAA | |
| 15 | 284 | | M13F-GTGCCTGGCCAGGGGTTGTGC | M13R-ATAAAAGCCATCAGTATTGTAG | |
| 16 | 309 | | M13F-TTTATTGTGTGATACATGTTTACT | M13R-AAAGAGGGATGAGGGAATAC | |
| 17 | 304 | | M13F-GTTGAATTCAGTATCATCCTATG | M13R-AAGTCACAGACTACACAGAAAC | |
| 18-1 | 259 | | M13F-CTTGTTTAAACAGTGGAATTCTA | M13R-ATCTGTAAGTTCAATAATGGCC | |
| 18-2 | 269 | | M13F-CAATAAAACTAGTAGTGCAGATA | M13R-GAATTTAACTGAATCAATGACTG | |
| 19-1 | 207 | | M13F-GGCAGTTCTAGAAGAATGAAAAC | M13R-ACATTTCCTCCATCACTGAAAAG | |
| 19-2 | 192 | | M13F-ATTCTTTCCTGACCCTAGACCTT | M13R-CCGAAACTCCATCTCAAACAAAC | |
| 20-1 | 396 | | M13F-CTCAGCCTCCCAAAGTTCTG | M13R-TTGTTGGGCCTCCACATATT | |
| 20-2 |  | | M13F-TCGCAATGAAAGAGAGGAAGA | M13R-TGTCCCTTGTTGCTATTCTTTGT | |
| 21 | 247 | | M13F-TGGGTGTTTTATGCTTGGTTC | M13R-TCATCAAGCCTCATTATATGTCC | |
| 22 | 302 | | M13F-TGTTCTGATTGCTTTTTATTCCA | M13R-GTTAGTAAGGTCATTTTTTAAGTT | |
| 23 | 273 | | M13F-TTTAAATGATAATCACTTCTTCC | M13R-TCCATAAACTAACAAGCACTTAT | |
| 24-1 | 155 | | M13F-TTTATGGAATCTCCATATGTTGA | M13R-CCTATTAGGTCCACCTCAG | |
| 24-2 | 174 | | M13F-CAGCAAATTTTTAGATCCAGAC | M13R-CTGGTAGCTCCAACTAATCAT | |
| 25 | 344 | | M13F-CTTAAAATTCATCTAACACATCTA | M13R-AAAAATACCAAAATGTGTGGTGA | |
| 26 | 277 | | M13F-GGAAACATAAATATGTGGGTTTG | M13R-GTATACAACAGAATATACGATGG | |
| 27-1 | 293 | | M13F-AGACTGTGTGTAATATTTGCGT | M13R-GTCTACTCAAGAAATCCAAGG | |
| 27-2 | 296 | | M13F-AGATTGATGACCAAAAGAACTG | M13R-TGAACCAGACAAAAGAGCTTG | |
| 27-3 | 299 | | M13F-TTCAATAGCTGACGAAGAACTT | M13R-TATAAACTGGAAAGGTTAAGCG | |
| *^*^M13F:* | | | *TGTAAAACGACGGCCAGT* | |  |
| *^#^M13R:* | | | *CAGGAAACAGCTATGACC* | |  |
