# Supplementary material for: Identification of BRCA1/2 Founder Mutations in Southern Chinese Breast Cancer Patients Using Gene Sequencing and High Resolution DNA Melting Analysis
Source: PLoS One. 2012 Sep 7;7(9):e43994. doi: 10.1371/journal.pone.0043994 (PMC3436879; doi:10.1371/journal.pone.0043994)
Supplement: Table S3 — Sequences of PCR primers for microsatellite polymorphic markers. (DOC) [file pone.0043994.s003.doc]

**Table S3** Sequences of PCR primers for microsatellite polymorphic markers

| **Name** | **Forward Primer Sequence (5' to 3')** | **Reverse Primer Sequence (5' to 3')** |
| --- | --- | --- |
| D13S1304 | ACCAGCCTTTGCTTAGGA | ACATTCTAGTGCTACAGGGTACTC |
| D13S217 | ATGCTGGGATCACAGGC | AACCTGGTGGACTTTTGCT |
| D13S289 | CTGGTTGAGCGGCATT | TGCAGCCTGGATGACA |
| D13S1699 | AGACAGAGAATCTCAACTGG | TTTGATTTTCACAGCAGATG |
| D13S1698 | GTCCATACCACTAAGTCTGAC | AACCTCAGGCTAATAGTCTCA |
| D13S171 | CCTACCATTGACACTCTCAG | TAGGGCCATCCATTCT |
| D13S1695 | AGAATCATTGCCCTACTTA | GATAACTTACCAGCATGTGA |
| D13S267 | GGCCTGAAAGGTATCCTC | TCCCACCATAAGCACAAG |
| D17S855 | GGATGGCCTTTTAGAAAGTGG | ACACAGACTTGTCCTACTGCC |
| D17S1323 | TAGGAGATGGATTATTGGTG | TAGGAGATGGATTATTGGTG |
| D17S1322 | CTAGCCTGGGCAACAAACGA | AAGCAACTTTGCAATGAGTG |
| D17S1335 | TGTCTGTTCACCAGGGTCTG | GCAGGAAGCAGGAATGGAAC |
| D17S1185 | GGTGACAGAACAAGACTCCATC | GCTAAAAATACACGGATGG |
