# Supplementary material for: Identification of BRCA1/2 Founder Mutations in Southern Chinese Breast Cancer Patients Using Gene Sequencing and High Resolution DNA Melting Analysis
Source: PLoS One. 2012 Sep 7;7(9):e43994. doi: 10.1371/journal.pone.0043994 (PMC3436879; doi:10.1371/journal.pone.0043994)
Supplement: Table S4 — The breakdown of mutations and variants of BRCA1 and BRCA2 in the 24 patients in the blind validation. (DOC) [file pone.0043994.s004.doc]

**Table S4** The breakdown of mutations and variants of *BRCA1* and *BRCA2* in the 24 patients in the blind validation

| **Gene** | **Mutations** | **Number** | **Total true Positive** |
| --- | --- | --- | --- |
| *BRCA1* | *Deleterious mutation* |  | 120 |
|  | c.3214delC | 1 |  |
|  | c.470_471delCT | 1 |  |
|  | *Missense mutation* |  |  |
|  | c.114G>A | 3 |  |
|  | c.571G>A | 1 |  |
|  | c.4484+14A>G | 1 |  |
|  | c.5406+33A>T | 1 |  |
|  | *Common SNP* |  |  |
|  | c.2082C>T | 16 |  |
|  | c.2311T>C | 16 |  |
|  | c.2612C>T | 16 |  |
|  | c.3113A>G | 16 |  |
|  | c.3548A>G | 16 |  |
|  | c.4308T>C | 16 |  |
|  | c.4837A>G | 16 |  |
| *BRCA2* | *Deleterious mutation* |  | 76 |
|  | c.2808_2811delACAA | 1 |  |
|  | c.7409_7410insT | 1 |  |
|  | *Missense mutation* |  |  |
|  | c.14G>A | 4 |  |
|  | c.943T>A | 1 |  |
|  | c.2133C>T | 1 |  |
|  | c.3807T>C | 1 |  |
|  | c.5785A>G | 1 |  |
|  | c.7469T>C | 1 |  |
|  | c.9271G>A | 1 |  |
|  | *Common SNP* |  |  |
|  | c.865 | 5 |  |
|  | c.1114C>A | 20 |  |
|  | c.1365A>G | 5 |  |
|  | c.2229T>C | 5 |  |
|  | c.2971A>G | 5 |  |
|  | c.3396A>G | 12 |  |
|  | c.7242A>G | 12 |  |
