# Supplementary material for: Identification of BRCA1/2 Founder Mutations in Southern Chinese Breast Cancer Patients Using Gene Sequencing and High Resolution DNA Melting Analysis
Source: PLoS One. 2012 Sep 7;7(9):e43994. doi: 10.1371/journal.pone.0043994 (PMC3436879; doi:10.1371/journal.pone.0043994)
Supplement: Table S5 — Genotype of carriers with BRCA1 or BRCA2 founder mutations and family members without mutations. (DOC) [file pone.0043994.s005.doc]

**Table S5.1** Genotype of carriers with *BRCA1* c.3342_3345delAGAA mutation and family members without mutation. No founder effect can be demonstrated by using PHASE program.

| **Groups** | **Case no.** | **Markers** | | | |
| --- | --- | --- | --- | --- | --- |
|  |  | **D17S855** | **D17S1185** | **D17S1322** | **D17S1323** |
| Family 1 | ***HKSH2101*** | **141**/147 | **218**/210 | **123**/114 | **159**/141 |
|  | HKSH2102 | **141**/**141** | **218**/206 | 114/117 | **159**/157 |
|  | ***HKSH2103*** | **141**/**141** | **218**/**218** | **123**/117 | **159**/157 |
| Family 2 | ***TWH42901*** | **141**/145 | **218**/222 | **123**/117 | **159**/151 |
|  | ***TWH42902*** | **141**/**141** | **218**/**218** | **123**/120 | **159**/161 |
| Unrelated Controls | N1 | 145/147 | 214/214 | 117/117 | 151/153 |
|  | N2 | 143/151 | 214/218 | 117/120 | 153/159 |
|  | N3 | 147/149 | 206/222 | 117/117 | 151/153 |
|  | N4 | 147/151 | 210/222 | 117/123 | 151/153 |
|  | N5 | 143/147 | 214/218 | 111/126 | 153/163 |
|  | N6 | 141/149 | 218/218 | 117/126 | 155/159 |
|  | N7 | 141/143 | 206/222 | 120/123 | 155/157 |
|  | N8 | 147/147 | 218/234 | 117/117 | 153/155 |
|  | N9 | 143/143 | 214/214 | 120/120 | 157/159 |
|  | N10 | 145/149 | 214/222 | 117/120 | 151/153 |
|  | N11 | 139/139 | 206/214 | 120/123 | 157/159 |
|  | N12 | 143/147 | 214/214 | 117/120 | 153/157 |
|  | N13 | 147/147 | 214/214 | 117/117 | 153/155 |
|  | N14 | 139/143 | 210/218 | 117/120 | 153/157 |
|  | N15 | 141/147 | 214/218 | 123/126 | 155/159 |
|  | N16 | 139/147 | 214/222 | 117/120 | 149/153 |
|  | N17 | 143/143 | 218/222 | 117/123 | 153/157 |
|  | N18 | 143/145 | 214/218 | 120/123 | 153/157 |
|  | N19 | 147/147 | 214/218 | 120/126 | 153/159 |
|  | N20 | 141/147 | 214/218 | 117/129 | 153/155 |
|  | N21 | 143/149 | 210/210 | 117/123 | 153/159 |
|  | N22 | 143/151 | 218/222 | 114/120 | 155/157 |
|  | N23 | 143/147 | 214/218 | 117/120 | 153/157 |
|  | N24 | 139/141 | 206/218 | 117/120 | 157/159 |
|  | N25 | 139/147 | 206/222 | 114/117 | 153/163 |
|  | N26 | 147/151 | 222/226 | 114/123 | 151/153 |
|  | N27 | 143/145 | 214/218 | 114/120 | 153/157 |
|  | N28 | 145/147 | 214/226 | 117/120 | 151/153 |
|  | N29 | 143/147 | 218/226 | 114/120 | 153/159 |
|  | N30 | 141/149 | 214/222 | 114/117 | 151/153 |
|  | N31 | 147/149 | 206/218 | 114/117 | 151/153 |
|  | N32 | 141/147 | 214/222 | 117/120 | 155/157 |
|  | N33 | 139/145 | 214/222 | 114/117 | 153/161 |
|  | N34 | 147/149 | 206/210 | 117/117 | 151/153 |
|  | N35 | 143/147 | 210/222 | 117/120 | 145/153 |
|  | N36 | 143/147 | 210/214 | 117/120 | 153/159 |
|  | N37 | 141/143 | 210/230 | 120/123 | 157/159 |
|  | N38 | 141/143 | 218/222 | 120/123 | 159/161 |
|  | N39 | 145/145 | 210/214 | 108/114 | 141/153 |
|  | N42 | 145/145 | 214/214 | 117/120 | 153/157 |
|  | N47 | 145/147 | 206/222 | 117/120 | 153/159 |
|  | N48 | 141/143 | 210/214 | 111/120 | 157/159 |
|  | N49 | 141/145 | 222/222 | 120/123 | 157/159 |
|  | N51 | 145/147 | 206/210 | 117/120 | 151/153 |
|  | N52 | 143/143 | 206/218 | 117/123 | 153/159 |
|  | NC268 | 143/147 | 206/214 | 117/120 | 153/157 |
|  | NC176 | 147/147 | 206/210 | 117/123 | 153/157 |
|  | NC181 | 145/149 | 206/214 | 114/120 | 151/153 |
|  | NC182 | 143/149 | 214/218 | 108/120 | 153/161 |
|  | NC247 | 147/147 | 210/222 | 114/117 | 141/153 |

*Unrelated probands highlighted in yellow; BRCA mutation positive cases are in bold and italic; Common genotypes are in bold.*

**Table S5.2** Genotype of carriers with *BRCA1* c.5406+1_5406+3_delGTA mutation and family members without mutation. No founder effect can be demonstrated by using PHASE program.

| **Groups** | **Case no.** | **Markers** | | | | | |
| --- | --- | --- | --- | --- | --- | --- | --- |
|  |  | **D17S855** | **D17S1185** | **D17S1322** | **D17S1323** | **D17S1335** | **D17S791** |
| Family 1 | ***HKSH7001*** | **145**/149 | **218**/214 | **114**/120 | **153**/161 | **167**/164 | **163**/177 |
|  | ***HKSH7002*** | **145**/151 | **218**/214 | **114**/117 | **153**/151 | **167**/164 | **163**/181 |
| Family 2 | ***TWH7401*** | **145**/139 | **218**/214 | **114**/120 | **153**/159 | **167**/164 | **163**/181 |
| Unrelated Controls | N1 | 145/147 | 214/214 | 117/117 | 151/153 | 164/164 | 177/177 |
|  | N2 | 143/151 | 214/218 | 117/120 | 153/159 | 164/168 | 165/187 |
|  | N3 | 147/149 | 206/222 | 117/117 | 151/153 | 164/164 | 163/163 |
|  | N4 | 147/151 | 210/222 | 117/123 | 151/153 | 164/164 | 165/189 |
|  | N5 | 143/147 | 214/218 | 111/126 | 153/163 | 164/164 | 175/175 |
|  | N6 | 141/149 | 218/218 | 117/126 | 155/159 | 164/168 | 163/177 |
|  | N7 | 141/143 | 206/222 | 120/123 | 155/157 | 164/164 | 185/187 |
|  | N8 | 147/147 | 218/234 | 117/117 | 153/155 | 164/168 | 175/189 |
|  | N9 | 143/143 | 214/214 | 120/120 | 157/159 | 164/168 | 177/191 |
|  | N10 | 145/149 | 214/222 | 117/120 | 151/153 | 164/168 | 163/189 |
|  | N11 | 139/139 | 206/214 | 120/123 | 157/159 | 160/164 | 175/177 |
|  | N12 | 143/147 | 214/214 | 117/120 | 153/157 | 164/164 | 175/175 |
|  | N13 | 147/147 | 214/214 | 117/117 | 153/155 | 164/164 | 163/185 |
|  | N14 | 139/143 | 210/218 | 117/120 | 153/157 | 164/168 | 163/179 |
|  | N15 | 141/147 | 214/218 | 123/126 | 155/159 | 164/168 | 175/187 |
|  | N16 | 139/147 | 214/222 | 117/120 | 149/153 | 164/164 | 163/175 |
|  | N17 | 143/143 | 218/222 | 117/123 | 153/157 | 164/164 | 175/181 |
|  | N18 | 143/145 | 214/218 | 120/123 | 153/157 | 164/164 | 163/175 |
|  | N19 | 147/147 | 214/218 | 120/126 | 153/159 | 164/164 | 163/187 |
|  | N20 | 141/147 | 214/218 | 117/129 | 153/155 | 164/164 | 163/181 |
|  | N21 | 143/149 | 210/210 | 117/123 | 153/159 | 164/164 | 163/175 |
|  | N22 | 143/151 | 218/222 | 114/120 | 155/157 | 164/168 | 175/177 |
|  | N23 | 143/147 | 214/218 | 117/120 | 153/157 | 164/164 | 181/185 |
|  | N24 | 139/141 | 206/218 | 117/120 | 157/159 | 164/164 | 163/187 |
|  | N25 | 139/147 | 206/222 | 114/117 | 153/163 | 164/164 | 163/179 |
|  | N26 | 147/151 | 222/226 | 114/123 | 151/153 | 164/164 | 175/179 |
|  | N27 | 143/145 | 214/218 | 114/120 | 153/157 | 164/164 | 181/183 |
|  | N28 | 145/147 | 214/226 | 117/120 | 151/153 | 160/164 | 175/189 |
|  | N29 | 143/147 | 218/226 | 114/120 | 153/159 | 164/164 | 163/163 |
|  | N30 | 141/149 | 214/222 | 114/117 | 151/153 | 164/168 | 177/187 |
|  | N31 | 147/149 | 206/218 | 114/117 | 151/153 | 164/164 | 185/187 |
|  | N32 | 141/147 | 214/222 | 117/120 | 155/157 | 164/164 | 179/179 |
|  | N33 | 139/145 | 214/222 | 114/117 | 153/161 | 164/164 | 163/179 |
|  | N34 | 147/149 | 206/210 | 117/117 | 151/153 | 164/164 | 175/179 |
|  | N35 | 143/147 | 210/222 | 117/120 | 145/153 | 164/164 | 163/163 |
|  | N36 | 143/147 | 210/214 | 117/120 | 153/159 | 164/164 | 175/175 |
|  | N37 | 141/143 | 210/230 | 120/123 | 157/159 | 164/164 | 177/185 |
|  | N38 | 141/143 | 218/222 | 120/123 | 159/161 | 164/164 | 177/177 |
|  | N39 | 145/145 | 210/214 | 108/114 | 141/153 | 164/164 | 163/191 |
|  | N42 | 145/145 | 214/214 | 117/120 | 153/157 | 164/167 | 183/189 |
|  | N47 | 145/147 | 206/222 | 117/120 | 153/159 | 164/164 | 177/191 |
|  | N48 | 141/143 | 210/214 | 111/120 | 157/159 | 164/164 | 177/185 |
|  | N49 | 141/145 | 222/222 | 120/123 | 157/159 | 164/164 | 163/189 |
|  | N51 | 145/147 | 206/210 | 117/120 | 151/153 | 164/164 | 179/183 |
|  | N52 | 143/143 | 206/218 | 117/123 | 153/159 | 164/164 | 163/179 |
|  | NC268 | 143/147 | 206/214 | 117/120 | 153/157 | 164/167 | 163/187 |
|  | NC176 | 147/147 | 206/210 | 117/123 | 153/157 | 164/164 | 163/189 |
|  | NC181 | 145/149 | 206/214 | 114/120 | 151/153 | 164/164 | 175/181 |
|  | NC182 | 143/149 | 214/218 | 108/120 | 153/161 | 164/167 | 163/175 |
|  | NC247 | 147/147 | 210/222 | 114/117 | 141/153 | 164/167 | 175/187 |

*Unrelated probands highlighted in yellow; BRCA mutation positive cases are in bold and italic; Common genotypes are in bold.*

**Table S5.3** Genotype of carriers with *BRCA1* c.981_982delAT mutation and family members without mutation. Founder effect can be demonstrated by using PHASE program.

| **Groups** | **Case no.** | **Markers** | | | | | |
| --- | --- | --- | --- | --- | --- | --- | --- |
|  |  | **D17S855** | **D17S1185** | **D17S1322** | **D17S1323** | **D17S1335** | **D17S791** |
| Family 1 | ***TWH30501*** | **143**/139 | **210**/218 | **120**/117 | **157**/161 | **164/164** | **167**/179 |
|  | ***TWH30501-1*** | **143**/149 | **210**/218 | **120**/117 | **157**/153 | **164**/167 | **167**/177 |
|  | ***TWH30501-2*** | **143**/147 | **210**/218 | **120**/117 | **157**/153 | **164**/160 | **167**/163 |
|  | TWH30521 | 139/147 | **210**/218 | **120**/117 | 153/161 | **164/164** | 175/179 |
|  | TWH30502 | 139/149 | 214/218 | **120**/117 | 153/161 | **164**/167 | 177/179 |
|  | TWH30503 | 139/149 | 214/218 | **120**/117 | 153/161 | **164**/167 | 177/179 |
|  | ***TWH30504*** | **143**/147 | **210**/**210** | **120**/117 | **157**/153 | **164/164** | **167**/175 |
|  | TWH30505 | 147/149 | **210**/214 | 114/117 | 151/153 | **164**/167 | 175/177 |
| Family 2 | ***FP6801*** | **143**/147 | **210**/214 | **120**/114 | **157**/153 | **164/164** | **167**/163 |
| Unrelated Controls | N1 | 145/147 | 214/214 | 117/117 | 151/153 | 164/164 | 177/177 |
|  | N2 | 143/151 | 214/218 | 117/120 | 153/159 | 164/168 | 165/187 |
|  | N3 | 147/149 | 206/222 | 117/117 | 151/153 | 164/164 | 163/163 |
|  | N4 | 147/151 | 210/222 | 117/123 | 151/153 | 164/164 | 165/189 |
|  | N5 | 143/147 | 214/218 | 111/126 | 153/163 | 164/164 | 175/175 |
|  | N6 | 141/149 | 218/218 | 117/126 | 155/159 | 164/168 | 163/177 |
|  | N7 | 141/143 | 206/222 | 120/123 | 155/157 | 164/164 | 185/187 |
|  | N8 | 147/147 | 218/234 | 117/117 | 153/155 | 164/168 | 175/189 |
|  | N9 | 143/143 | 214/214 | 120/120 | 157/159 | 164/168 | 177/191 |
|  | N10 | 145/149 | 214/222 | 117/120 | 151/153 | 164/168 | 163/189 |
|  | N11 | 139/139 | 206/214 | 120/123 | 157/159 | 160/164 | 175/177 |
|  | N12 | 143/147 | 214/214 | 117/120 | 153/157 | 164/164 | 175/175 |
|  | N13 | 147/147 | 214/214 | 117/117 | 153/155 | 164/164 | 163/185 |
|  | N14 | 139/143 | 210/218 | 117/120 | 153/157 | 164/168 | 163/179 |
|  | N15 | 141/147 | 214/218 | 123/126 | 155/159 | 164/168 | 175/187 |
|  | N16 | 139/147 | 214/222 | 123/126 | 149/153 | 164/164 | 163/175 |
|  | N17 | 143/143 | 218/222 | 117/123 | 153/157 | 164/164 | 175/181 |
|  | N18 | 143/145 | 214/218 | 120/123 | 153/157 | 164/164 | 163/175 |
|  | N19 | 147/147 | 214/218 | 120/126 | 153/159 | 164/164 | 163/187 |
|  | N20 | 141/147 | 214/218 | 117/129 | 153/155 | 164/164 | 163/181 |
|  | N21 | 143/149 | 210/210 | 117/123 | 153/159 | 164/164 | 163/175 |
|  | N22 | 143/151 | 218/222 | 114/120 | 155/157 | 164/168 | 175/177 |
|  | N23 | 143/147 | 214/218 | 117/120 | 153/157 | 164/164 | 181/185 |
|  | N24 | 139/141 | 206/218 | 117/120 | 157/159 | 164/164 | 163/187 |
|  | N25 | 139/147 | 206/222 | 114/117 | 153/163 | 164/164 | 163/179 |
|  | N26 | 147/151 | 222/226 | 114/123 | 151/153 | 164/164 | 175/179 |
|  | N27 | 143/145 | 214/218 | 114/120 | 153/157 | 164/164 | 181/183 |
|  | N28 | 145/147 | 214/226 | 117/120 | 151/153 | 160/164 | 175/189 |
|  | N29 | 143/147 | 218/226 | 114/120 | 153/159 | 164/164 | 163/163 |
|  | N30 | 141/149 | 214/222 | 114/117 | 151/153 | 164/168 | 177/187 |
|  | N31 | 147/149 | 206/218 | 114/117 | 151/153 | 164/164 | 185/187 |
|  | N32 | 141/147 | 214/222 | 117/120 | 155/157 | 164/164 | 179/179 |
|  | N33 | 139/145 | 214/222 | 114/117 | 153/161 | 164/164 | 163/179 |
|  | N34 | 147/149 | 206/210 | 117/117 | 151/153 | 164/164 | 175/179 |
|  | N35 | 143/147 | 210/222 | 117/120 | 145/153 | 164/164 | 163/163 |
|  | N36 | 143/147 | 210/214 | 117/120 | 153/159 | 164/164 | 175/175 |
|  | N37 | 141/143 | 210/230 | 120/123 | 157/159 | 164/164 | 177/185 |
|  | N38 | 141/143 | 218/222 | 120/123 | 159/161 | 164/164 | 177/177 |
|  | N39 | 145/145 | 210/214 | 108/114 | 141/153 | 164/164 | 163/191 |
|  | N42 | 145/145 | 214/214 | 117/120 | 153/157 | 164/167 | 183/189 |
|  | N47 | 145/147 | 206/222 | 117/120 | 153/159 | 164/164 | 177/191 |
|  | N48 | 141/143 | 210/214 | 111/120 | 157/159 | 164/164 | 177/185 |
|  | N49 | 141/145 | 222/222 | 120/123 | 157/159 | 164/164 | 163/189 |
|  | N51 | 145/147 | 206/210 | 117/123 | 151/153 | 164/164 | 179/183 |
|  | N52 | 143/143 | 206/218 | 117/123 | 153/159 | 164/164 | 163/179 |
|  | NC268 | 143/147 | 206/214 | 117/120 | 153/157 | 164/167 | 163/187 |
|  | NC176 | 147/147 | 206/210 | 117/123 | 153/157 | 164/164 | 163/189 |
|  | NC181 | 145/149 | 206/214 | 114/120 | 151/153 | 164/164 | 175/181 |
|  | NC182 | 143/149 | 214/218 | 108/120 | 153/161 | 164/167 | 163/175 |
|  | NC247 | 147/147 | 210/222 | 114/117 | 141/153 | 164/167 | 175/187 |

*Unrelated probands highlighted in yellow; BRCA mutation positive cases are in bold and italic; Common genotypes are in bold.*

**Haplotype analysis of the *BRCA1* c.981_982delAT mutation carriers**

|  | **1** |  |  |  | **2** |  |  |  | **3** |  |  |  | **4** |  |  |  | **5** |  |  |  |
| --- | --- | --- | --- | --- | --- | --- | --- | --- | --- | --- | --- | --- | --- | --- | --- | --- | --- | --- | --- | --- |
| 164 |  | **164** |  | 167 |  | **164** |  | 160 |  | **164** |  | 164 |  | **164** |  | 164 |  | **164** |  | D17S1335 |
| 218 |  | **210** |  | 218 |  | **210** |  | 218 |  | **210** |  | 210 |  | **210** |  | 214 |  | **210** |  | D17S1185 |
| 139 |  | **143** |  | 149 |  | **143** |  | 147 |  | **143** |  | 147 |  | **143** |  | 147 |  | **143** |  | D17S855 |
| 117 |  | **120** |  | 117 |  | **120** |  | 117 |  | **120** |  | 117 |  | **120** |  | 114 |  | **120** |  | D17S1322 |
| 161 |  | **157** |  | 153 |  | **157** |  | 153 |  | **157** |  | 153 |  | **157** |  | 153 |  | **157** |  | D17S1323 |
| 179 |  | **167** |  | 177 |  | **167** |  | 163 |  | **167** |  | 175 |  | **167** |  | 163 |  | **167** |  | D17S791 |

| Mutation carriers | |
| --- | --- |
| 1 | TWH30501 |
| 2 | TWH30501-1 |
| 3 | TWH30501-2 |
| 4 | TWH30504 |
| 5 | FP6801 |

**Table S5.4** Genotype of carriers with *BRCA2* c.3109C>T mutation and family members without mutation. Founder effect can be demonstrated by using PHASE program.

| **Groups** | **Case no.** | **Markers** | | | |
| --- | --- | --- | --- | --- | --- |
|  |  | **D13S289** | **D13S1695** | **D13S1698** | **D13S1699** |
| Family 1 | ***HKSH3401*** | **262/**274 | **241/**253 | **163/**167 | **156/**153 |
| Family 2 | ***HKSH5601*** | **262**/274 | **241**/257 | **163/163** | **156**/156 |
| Family 3 | ***TWH11401*** | **262/262** | **241**/255 | **163**/167 | **156**/153 |
|  | TWH11401-1 | 264/274 | 255/255 | 167/167 | 153/153 |
|  | TWH11401-2 | 264/264 | 255/263 | 165/167 | 153/153 |
| Family 4 | ***TWH2901*** | **262/262** | **241**/255 | **163/163** | **156/156** |
| Family 5 | ***TWH4001*** | **262/262** | **241**/255 | **163**/165 | **156**/153 |
|  | ***TWH4001-1*** | **262**/274 | **241**/255 | **163**/165 | **156**/153 |
|  | TWH4001-2 | **262**/274 | 255/255 | 165/165 | 153/153 |
| Family 6 | ***TWH16901*** | **262**/264 | **241/241** | **163/163** | **156**/153 |
|  | TWH16901-1 | 264/272 | **241/241** | **163/163** | 153/153 |
|  | TWH16902 | 266/274 | 253/253 | **163/163** | **156**/153 |
| Unrelated Controls | N1 | 262/276 | 241/255 | 159/165 | 153/153 |
|  | N2 | 262/262 | 253/257 | 163/173 | 153/153 |
|  | N3 | 262/266 | 253/253 | 163/163 | 153/156 |
|  | N4 | 262/262 | 241/255 | 163/167 | 153/153 |
|  | N5 | 266/274 | 253/263 | 159/163 | 153/156 |
|  | N6 | 262/274 | 253/253 | 163/163 | 153/153 |
|  | N7 | 266/274 | 253/253 | 165/171 | 153/153 |
|  | N8 | 262/262 | 253/253 | 165/165 | 156/156 |
|  | N9 | 262/274 | 253/253 | 163/163 | 153/156 |
|  | N10 | 262/276 | 241/253 | 163/167 | 153/160 |
|  | N11 | 262/262 | 255/255 | 163/163 | 153/153 |
|  | N12 | 262/262 | 241/257 | 163/175 | 152/161 |
|  | N13 | 274/274 | 255/255 | 163/163 | 156/156 |
|  | N14 | 272/272 | 241/255 | 163/167 | 153/153 |
|  | N16 | 272/272 | 243/255 | 163/167 | 153/153 |
|  | N17 | 262/274 | 253/257 | 163/167 | 153/153 |
|  | N18 | 262/262 | 241/253 | 165/165 | 153/153 |
|  | N19 | 264/272 | 255/255 | 159/163 | 153/153 |
|  | N20 | 264/272 | 241/257 | 159/163 | 153/156 |
|  | N21 | 274/274 | 253/253 | 161/165 | 153/156 |
|  | N22 | 262/266 | 241/253 | 163/163 | 153/153 |
|  | N23 | 264/274 | 241/253 | 163/163 | 153/153 |
|  | N24 | 262/262 | 253/253 | 165/165 | 153/153 |
|  | N25 | 266/274 | 255/259 | 159/175 | 153/153 |
|  | N26 | 262/274 | 251/251 | 165/165 | 153/153 |
|  | N27 | 262/262 | 241/259 | 165/173 | 153/156 |
|  | N28 | 262/272 | 251/263 | 159/165 | 153/153 |
|  | N29 | 264/274 | 241/261 | 163/163 | 153/156 |
|  | N30 | 272/272 | 253/253 | 163/167 | 153/153 |
|  | N31 | 270/270 | 255/255 | 163/169 | 153/156 |
|  | N32 | 262/272 | 241/253 | 163/165 | 153/160 |
|  | N33 | 262/272 | 253/253 | 165/169 | 153/153 |
|  | N34 | 262/272 | 253/253 | 163/173 | 152/156 |
|  | N35 | 262/272 | 241/253 | 165/169 | 153/153 |
|  | N36 | 262/262 | 241/257 | 163/163 | 153/153 |
|  | N37 | 262/262 | 241/241 | 163/167 | 153/153 |
|  | N38 | 274/274 | 243/255 | 165/165 | 153/153 |
|  | N39 | 264/264 | 253/253 | 159/165 | 153/153 |
|  | N41 | 262/274 | 241/263 | 167/167 | 153/156 |
|  | N42 | 262/266 | 241/253 | 165/165 | 153/153 |
|  | N45 | 264/270 | 241/255 | 159/163 | 153/156 |
|  | N47 | 262/272 | 253/253 | 163/163 | 156/156 |
|  | N48 | 262/266 | 243/255 | 165/169 | 156/156 |
|  | N49 | 262/272 | 241/253 | 163/163 | 153/153 |
|  | N53 | 272/272 | 255/255 | 165/165 | 153/153 |
|  | NC176 | 262/262 | 255/255 | 163/163 | 153/153 |
|  | NC181 | 266/274 | 253/253 | 159/167 | 153/153 |
|  | NC182 | 264/272 | 253/253 | 165/165 | 153/156 |
|  | NC247 | 262/262 | 241/259 | 163/167 | 153/153 |
|  | NC268 | 272/272 | 241/259 | 163/167 | 153/156 |

*Unrelated probands highlighted in yellow; BRCA mutation positive cases are in bold and italic; Common genotypes are in bold.*

**Haplotype analysis of the *BRCA2* c.3109C>T mutation carriers**

|  | **1** |  |  |  | **2** |  |  |  | **3** |  |  |  | **4** |  |  |  | **5** |  |  |  | **6** |  |  |  | **7** |  |  |  |
| --- | --- | --- | --- | --- | --- | --- | --- | --- | --- | --- | --- | --- | --- | --- | --- | --- | --- | --- | --- | --- | --- | --- | --- | --- | --- | --- | --- | --- |
| 274 |  | **262** |  | 274 |  | **262** |  | 262 |  | **262** |  | 262 |  | **262** |  | 262 |  | **262** |  | 274 |  | **262** |  | 264 |  | **262** |  | D17S289 |
| 253 |  | **241** |  | 257 |  | **241** |  | 255 |  | **241** |  | 255 |  | **241** |  | 255 |  | **241** |  | 255 |  | **241** |  | 241 |  | **241** |  | D17S1695 |
| 167 |  | **163** |  | 163 |  | **163** |  | 167 |  | **163** |  | 163 |  | **163** |  | 165 |  | **163** |  | 165 |  | **163** |  | 163 |  | **163** |  | D17S1698 |
| 153 |  | **156** |  | 156 |  | **156** |  | 153 |  | **156** |  | 156 |  | **156** |  | 153 |  | **156** |  | 153 |  | **156** |  | 153 |  | **156** |  | D17S1699 |

| Mutation carriers | |
| --- | --- |
| 1 | HKSH3401 |
| 2 | HKSH5601 |
| 3 | TWH11401 |
| 4 | TWH2901 |
| 5 | TWH4001 |
| 6 | TWH4001-1 |
| 7 | TWH16901 |

**Table S5.5** Genotype of carriers with *BRCA2* c.7436_7805del370 mutation and family members without mutation. Founder effect can be demonstrated by using PHASE program.

| **Groups** | **Case no.** | **Markers** | | |
| --- | --- | --- | --- | --- |
|  |  | **D13S289** | **D13S1698** | **D13S1699** |
| Family 1 | ***PMH1931*** | **262**/272 | **171/**165 | **152/152** |
|  | ***PMH1901-1*** | **262**/266 | **171**/161 | **152/152** |
|  | PMH1901-2 | **262**/272 | 163/165 | **152**/156 |
|  | ***PMH1901-3*** | **262**/266 | **171**/161 | **152/152** |
|  | ***PMH1941-1*** | **262**/274 | **171**/159 | **152/152** |
|  | ***PMH1941-2*** | **262**/274 | **171**/163 | **152/152** |
| Family 2 | ***HKSH9601*** | **262/262** | **171/**163 | **152/152** |
| Unrelated Controls | N1 | 262/276 | 159/165 | 153/153 |
|  | N2 | 262/262 | 163/173 | 153/153 |
|  | N3 | 262/266 | 163/163 | 153/156 |
|  | N4 | 262/262 | 163/167 | 153/153 |
|  | N5 | 266/274 | 159/163 | 153/156 |
|  | N6 | 262/274 | 163/163 | 153/153 |
|  | N7 | 266/274 | 165/171 | 153/153 |
|  | N8 | 262/262 | 165/165 | 156/156 |
|  | N9 | 262/274 | 163/163 | 153/156 |
|  | N10 | 262/276 | 163/167 | 153/160 |
|  | N11 | 262/262 | 163/163 | 153/153 |
|  | N12 | 262/262 | 163/175 | 152/161 |
|  | N13 | 274/274 | 163/163 | 156/156 |
|  | N14 | 272/272 | 163/167 | 153/153 |
|  | N16 | 272/272 | 163/167 | 153/153 |
|  | N17 | 262/274 | 163/167 | 153/153 |
|  | N18 | 262/262 | 165/165 | 153/153 |
|  | N19 | 264/272 | 159/163 | 153/153 |
|  | N20 | 264/272 | 159/163 | 153/156 |
|  | N21 | 274/274 | 161/165 | 153/156 |
|  | N22 | 262/266 | 163/163 | 153/153 |
|  | N23 | 264/274 | 163/163 | 153/153 |
|  | N24 | 262/262 | 165/165 | 153/153 |
|  | N25 | 266/274 | 159/175 | 153/153 |
|  | N26 | 262/274 | 165/165 | 153/153 |
|  | N27 | 262/262 | 165/173 | 153/156 |
|  | N28 | 262/272 | 159/165 | 153/153 |
|  | N29 | 264/274 | 163/163 | 153/156 |
|  | N30 | 272/272 | 163/167 | 153/153 |
|  | N31 | 270/270 | 163/169 | 153/156 |
|  | N32 | 262/272 | 163/165 | 153/160 |
|  | N33 | 262/272 | 165/169 | 153/153 |
|  | N34 | 262/272 | 163/173 | 152/156 |
|  | N35 | 262/272 | 165/169 | 153/153 |
|  | N36 | 262/262 | 163/163 | 153/153 |
|  | N37 | 262/262 | 163/167 | 153/153 |
|  | N38 | 274/274 | 165/165 | 153/153 |
|  | N39 | 264/264 | 159/165 | 153/153 |
|  | N41 | 262/274 | 167/167 | 153/156 |
|  | N42 | 262/266 | 165/165 | 153/153 |
|  | N45 | 264/270 | 159/163 | 153/156 |
|  | N47 | 262/272 | 163/163 | 156/156 |
|  | N48 | 262/266 | 165/169 | 156/156 |
|  | N49 | 262/272 | 163/163 | 153/153 |
|  | N53 | 272/272 | 165/165 | 153/153 |
|  | NC176 | 262/262 | 163/163 | 153/153 |
|  | NC181 | 266/274 | 159/167 | 153/153 |
|  | NC182 | 264/272 | 165/165 | 153/156 |
|  | NC247 | 262/262 | 163/167 | 153/153 |
|  | NC268 | 272/272 | 163/167 | 153/156 |

*Unrelated probands highlighted in yellow; BRCA mutation positive cases are in bold and italic; Common genotypes are in bold.*

**Haplotype analysis of the *BRCA2* c.7436_7805del370 mutation carriers**

|  | **1** |  |  |  | **2** |  |  |  | **3** |  |  |  | **4** |  |  |  | **5** |  |  |  | **6** |  |  |  |
| --- | --- | --- | --- | --- | --- | --- | --- | --- | --- | --- | --- | --- | --- | --- | --- | --- | --- | --- | --- | --- | --- | --- | --- | --- |
| 272 |  | **262** |  | 266 |  | **262** |  | 266 |  | **262** |  | 274 |  | **262** |  | 274 |  | **262** |  | 262 |  | **262** |  | D13S289 |
| 152 |  | **152** |  | 152 |  | **152** |  | 152 |  | **152** |  | 152 |  | **152** |  | 152 |  | **152** |  | 152 |  | **152** |  | D13S1699 |
| 165 |  | **171** |  | 161 |  | **171** |  | 161 |  | **171** |  | 159 |  | **171** |  | 163 |  | **171** |  | 163 |  | **171** |  | D13S1698 |

| Mutation carriers | |
| --- | --- |
| 1 | PMH1931 |
| 2 | PMH1901-1 |
| 3 | PMH1901-3 |
| 4 | PMH1941-1 |
| 5 | PMH1941-2 |
| 6 | HKSH9601 |

**Table S5.6** Genotype of carriers with *BRCA2* c.9097_9098insA mutation and family members without mutation. Founder effect can be demonstrated by using PHASE program.

| **Groups** | **Case no.** | **Markers** | | |
| --- | --- | --- | --- | --- |
|  |  | **D13S260** | **D13S289** | **D13S1695** |
| Family 1 | ***TWH40901*** | **174**/166 | **272/272** | **251/251** |
|  | ***TWH40902*** | **174**/**174** | **272**/262 | **251**/255 |
|  | ***TWH40903*** | **174**/**174** | **272**/262 | **251**/255 |
|  | TWH40904 | 162/166 | **272**/264 | **251**/257 |
|  | TWH40905 | 162/166 | **272**/264 | **251**/257 |
|  | TWH40906 | **174**/166 | 262/262 | 255/255 |
| Family 2 | ***NDH301*** | **174**/168 | **272/276** | **251/251** |
| Unrelated Controls | N1 | 166/166 | 262/276 | 241/255 |
|  | N2 | 168/172 | 262/262 | 253/257 |
|  | N3 | 168/176 | 262/266 | 253/253 |
|  | N4 | 168/172 | 262/262 | 241/255 |
|  | N5 | 166/166 | 266/274 | 253/263 |
|  | N6 | 168/176 | 262/274 | 253/253 |
|  | N7 | 174/174 | 266/274 | 253/253 |
|  | N8 | 176/176 | 262/262 | 253/253 |
|  | N9 | 172/172 | 262/274 | 253/253 |
|  | N10 | 168/174 | 262/276 | 241/253 |
|  | N11 | 166/166 | 262/262 | 255/255 |
|  | N12 | 172/172 | 262/262 | 241/257 |
|  | N13 | 174/174 | 274/274 | 255/255 |
|  | N14 | 168/168 | 272/272 | 241/255 |
|  | N16 | 166/174 | 272/272 | 243/255 |
|  | N17 | 166/174 | 262/274 | 253/257 |
|  | N18 | 168/168 | 262/262 | 241/253 |
|  | N19 | 168/172 | 264/272 | 255/255 |
|  | N20 | 166/166 | 264/272 | 241/257 |
|  | N21 | 168/168 | 274/274 | 253/253 |
|  | N22 | 168/170 | 262/266 | 241/253 |
|  | N23 | 168/178 | 264/274 | 241/253 |
|  | N24 | 166/166 | 262/262 | 253/253 |
|  | N25 | 174/174 | 266/274 | 255/259 |
|  | N26 | 168/174 | 262/274 | 251/251 |
|  | N27 | 166/172 | 262/262 | 241/259 |
|  | N28 | 168/168 | 262/272 | 251/263 |
|  | N29 | 168/168 | 264/274 | 241/261 |
|  | N30 | 172/172 | 272/272 | 253/253 |
|  | N31 | 168/176 | 270/270 | 255/255 |
|  | N32 | 166/166 | 262/272 | 241/253 |
|  | N33 | 168/178 | 262/272 | 253/253 |
|  | N34 | 168/168 | 262/272 | 253/253 |
|  | N35 | 168/168 | 262/272 | 241/253 |
|  | N36 | 168/178 | 262/262 | 241/257 |
|  | N37 | 168/178 | 262/262 | 241/241 |
|  | N38 | 168/176 | 274/274 | 243/255 |
|  | N39 | 166/172 | 264/264 | 253/253 |
|  | N41 | 172/182 | 262/274 | 241/263 |
|  | N42 | 166/168 | 262/266 | 241/253 |
|  | N45 | 166/166 | 264/270 | 241/255 |
|  | N47 | 172/178 | 262/272 | 253/253 |
|  | N48 | 178/178 | 262/266 | 243/255 |
|  | N49 | 176/176 | 262/272 | 241/253 |
|  | N53 | 168/174 | 272/272 | 255/255 |
|  | NC176 | 168/176 | 262/262 | 255/255 |
|  | NC181 | 168/172 | 266/274 | 253/253 |
|  | NC182 | 166/166 | 264/272 | 253/253 |
|  | NC247 | 168/178 | 262/262 | 241/259 |
|  | NC268 | 168/182 | 272/272 | 241/259 |

*Unrelated probands highlighted in yellow; BRCA mutation positive cases are in bold and italic; Common genotypes are in bold.*

**Haplotype analysis of the *BRCA2* c.9097_9098insA mutation carriers**

|  | **1** |  |  |  | **2** |  |  |  | **3** |  |  |  | **4** |  |  |  |
| --- | --- | --- | --- | --- | --- | --- | --- | --- | --- | --- | --- | --- | --- | --- | --- | --- |
| 272 |  | **272** |  | 262 |  | **272** |  | 262 |  | **272** |  | 276 |  | **272** |  | D13S289 |
| 166 |  | **174** |  | 174 |  | **174** |  | 174 |  | **174** |  | 168 |  | **174** |  | D13S260 |
| 251 |  | **251** |  | 255 |  | **251** |  | 255 |  | **251** |  | 251 |  | **251** |  | D13S1695 |

| Mutation carriers | |
| --- | --- |
| 1 | TWH40901 |
| 2 | TWH40902 |
| 3 | TWH40903 |
| 4 | NDH301 |
